# A Dual TLR7/TLR9 Inhibitor HJ901 Inhibits ABC-DLBCL Expressing the MyD88 L265P Mutation

**DOI:** 10.3389/fcell.2020.00262

**Published:** 2020-04-22

**Authors:** Beiying An, Shan Zhu, Tete Li, Jing Wu, Guoxia Zang, Xinping Lv, Yuan Qiao, Jing Huang, Yan Shao, Jiuwei Cui, Yong-Jun Liu, Jingtao Chen

**Affiliations:** ^1^Institute of Translational Medicine, The First Hospital of Jilin University, Changchun, China; ^2^Department of Clinical Laboratory, The First Hospital of Jilin University, Changchun, China; ^3^Changchun Huapu Biotechnology Co., Ltd., Changchun, China; ^4^Cancer Center, The First Hospital of Jilin University, Changchun, China

**Keywords:** ABC DLBCL, MyD88 mutation, TLR7, TLR9, signaling pathway, antagonist

## Abstract

Diffuse large B cell lymphoma (DLBCL) is associated with aggressive clinical cases and poor prognosis despite recent advances in disease treatment. In activated B-cell-like (ABC)-DLBCL, the most severe damaged signaling pathways converge to aberrantly activate the Toll-like receptor (TLR) 7/9/MyD88 pathways, leading to the avoidance of cell death and resistance to chemotherapy. A gain of function mutation in MyD88 (MyD88 L265P) enhanced the NF-κB and JAK-STAT signaling pathways and was associated with dysregulation of TLR signaling in the pathogenesis of ABC-DLBCL. Therefore, inhibition of the TLR signaling network may improve clinical outcomes. In this study, we designed a *de novo* synthesized oligodeoxynucleotide-based antagonist of TLR7 and TLR9, referred to as HJ901, which competitively binds to TLR7/9. We profiled HJ901 inhibition in various DLBCL cell lines and verified tumor suppression in a xenograft mouse model. We found that HJ901 treatment significantly reduced TLR7- and TLR9-mediated cell proliferation and cytokine production in a time- and dose-dependent manner in various DLBCL cell lines expressing the MyD88 L265P mutation. Moreover, HJ901 prevented tumor growth and downregulated the NF-κB and JAK2-STAT3 signaling pathways in a DLBCL xenograft mouse model with the MyD88 L265P mutation. These results reveal that the anti-tumor effects of the synthesized oligodeoxynucleotide-based antagonist, HJ901, which competitively binds to TLR7/9, may be associated with the downregulation of the NF-κB and JAK2-STAT3 signaling pathways and provide rationale for treating ABC-DLBCL patients with the MyD88 L265P mutation.

## Introduction

Toll-like receptors (TLRs) are pathogen-associated recognition receptors; there are eleven TLRs in humans. TLR activation is involved in the occurrence and development of various autoimmune diseases, such as rheumatoid arthritis, systemic lupus erythematosus, multiple sclerosis, and psoriasis (Marshak-Rothstein, 2006; Kawai and Akira, 2010). Among these receptors, TLR7 and TLR9 are found in endosomal compartments where they identify double-stranded viral RNA and bacterial and viral DNA containing unmethylated CpG (dinucleotide containing cytosine and guanine) motifs, respectively (Marshak-Rothstein, 2006; Kawai and Akira, 2010). Both TLR7 and TLR9 have been at the center of considerable controversy, as their manipulation can alleviate or exacerbate diseased conditions based on the circumstances. Indeed, antagonists of TLR7 and TLR9 are already used to remedy immune-mediated inflammatory disorders (Sun et al., 2007). Other reports reveal that TLR7 and TLR9 agonists effectively improve asthma and allergy in both humans and mice (Krieg, 2006; Camateros et al., 2007). However, targeting TLR7 and TLR9 for treatment of diffuse large B cell lymphoma (DLBCL) is poorly characterized.

Diffuse large B cell lymphoma is the most widespread form of non-Hodgkin lymphoma. Based on gene expression profiling, DLBCL is divided into three molecular subtypes: germinal-center B-cell-like DLBCL, primary mediastinal B-cell lymphoma, and activated B-cell-like DLBCL (ABC-DLBCL) (Staudt and Dave, 2005). ABC-DLBCL, which is characterized by constitutive activation of nuclear factor-kappa B (NF-κB), is thought to be the most aggressive subtype, and patients with ABC-DLBCL have a poorer clinical outcome with an approximated 40% overall survival rate compared to those with primary mediastinal B-cell lymphoma or germinal-center B-cell-like-DLBCL (Roschewski et al., 2014; Sehn and Gascoyne, 2015). NF-κB is transiently activated in various hematologic malignancies through multiple upstream signaling pathways, including TLR7/9 (Young et al., 2015; Monlish et al., 2016). TLR7/9 signaling acts through the adapter myeloid differentiation primary-response protein 88 (MyD88), which is essential for induction of NF-κB in several normal immune cell types (Isaza-Correa et al., 2014). Previous reports indicated that 39% of ABC-DLBCL cases exhibit a mutant isoform of MyD88 with a leucine to proline substitution at position 265 (L265P), similar to the resequencing of MyD88 in DLBCL clinical samples. This mutation induces NF-κB and JAK/STAT3 activation and promotes cell survival in ABC-DLBCL (Ding et al., 2008; Ngo et al., 2011). Moreover, in ABC-DLBCL, NF-κB enhances the secretion of interleukin (IL)-6 and/or IL-10, leading to the activation of JAK/STAT3 signaling, further enhancing NF-κB activity and its target gene expression (Lam et al., 2008a; Zegeye et al., 2018). Continuous stimulation of TLRs by microbial products constitutively activates the NF-κB and STAT3 transcription factors, which exert pro-cancerous activity via multiple effectors (Karin, 2006; Yu et al., 2007; Kawai and Akira, 2011). Accordingly, inhibition of the TLR7/9-MyD88-NF-κB or/and -JAK2/STAT3 signaling pathways may be useful for preventing and treating ABC-DLBCL.

Substantial evidence suggests that TLR7 and TLR9 antagonists exhibit potentially therapeutic effects in disease pathogenesis, including systemic lupus erythematosus, rheumatoid arthritis, Sjögren’s syndrome, multiple sclerosis, inflammatory bowel disease/colitis, and psoriasis (Sun et al., 2007). Furthermore, the suppressive DNA sequences/oligonucleotides (ODN) characterized by common oligos (dG) and long double-stranded DNA, ODN containing GpG or methylated CpG motifs, G-rich and GC-rich motifs, or phosphothioated ODN, downregulate TLR9- and/or TLR7-mediated immune responses (Chen et al., 2001; Zhu et al., 2002; Gursel et al., 2003; Lenert et al., 2003; Trieu et al., 2006). For example, ODN-A151, a novel ODN with mammal telomere-mimicking TTAGGG repeats that blocks TLR9 signaling, is required for the treatment of autoimmune and infectious diseases through the inhibition of AIM2 inflammasome activation (Kaminski et al., 2013). IMO-8400 contains an arabino-G/7-deaza-dG modification and 2′-*O*-methylribonucleotides as immune-stimulatory and -regulatory motifs, respectively, and is synthesized by oligonucleotide-based antagonists of TLR7, 8, and 9 (Kandimalla et al., 2013). Moreover, SAT05f, a human microsatellite DNA-mimicking ODN comprising CCT repeats, reduces D-galactosamine (D-GalN)/CpG ODN-induced lethal shock in mice and viral nucleic acids-stimulated IFN-α production *in vitro* by blocking TLR7/9 activation (Sun et al., 2010). More importantly, the structure of HJ901 is considerably different from the inhibitory ODNs reported previously (Lenert et al., 2001; Stunz et al., 2002; Gursel et al., 2003; Barrat et al., 2005). Most reported inhibitory ODNs are G-containing or poly G-containing or G-rich. Notably, HJ901 has no “G base,” which is composed of CCT repeats. In the current study, HJ901 was studied for its inhibitory effects on TLR7/9 activation and downregulation of the NF-κB and JAK2/STAT3 pathways, as well as its therapeutic effects on ABC-DLBCL with the MyD88 L265P mutation.

## Results

### HJ901 Selectively Inhibited SEAP Activation in HEK-Blue-hTLR7 or -hTLR9 Cells

**FIGURE 1:**
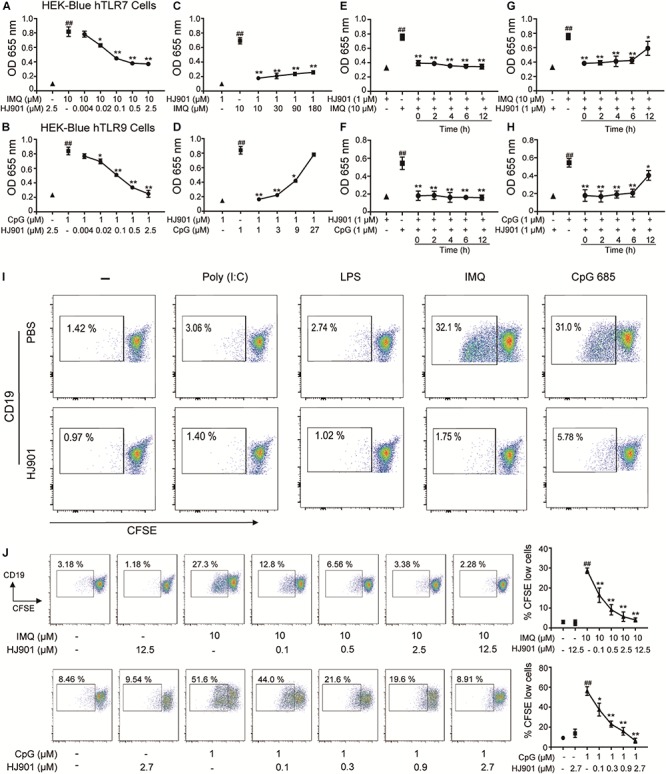
HJ901 selectively inhibits TLR7- and TLR9-mediated cell proliferation in HEK-Blue-hTLR7, HEK-Blue-hTLR9, or PBMCs cells. **(A,B)** HEK-Blue-hTLR7 or HEK-Blue-hTLR9 cells were cultured with 10 μM IMQ or 1 μM CpG 685 (CpG) in the presence or absence of different concentrations of HJ901 (0.004, 0.002, 0.1, 0.5, or 2.5 μM) for 24 h. **(C,D)** HEK-Blue-hTLR7 or HEK-Blue-hTLR9 cells were cultured with HJ901 in the presence or absence of different concentrations of IMQ (10, 30, 90, or 180 μM) or CpG (1, 3, 9, or 27 μM) for 24 h. **(E,F)** HEK-Blue-hTLR7 or HEK-Blue-hTLR9 cells were incubated with HJ901 for 0, 2, 4, 6, or 12 h and then treated with or without 10 μM IMQ or 1 μM CpG HJ901 for 24 h. **(G,H)** HEK-Blue-hTLR7 or HEK-Blue-hTLR9 cells were incubated with 10 μM IMQ or 1 μM CpG for 0, 2, 4, 6, or 12 h and then treated with or without HJ901 for 24 h, and the SEAP activity was determined. **(I)** Human PBMCs incorporated CFSE cultured with Poly (I: C), LPS, IMQ, or CpG in control ODN, and HJ901 cells for 5 days of treatment. **(J)** Human PBMCs incorporated CFSE cultured with IMQ or CpG in the presence or absence of different HJ901 concentrations for 5 days. Proliferation of CD19^+^ B cells was determined by CFSE dilution, which was assessed by flow cytometry. Quantification of three experiments is shown in the right panel. Similar results were obtained from three independent experiments. All data are presented as the means ± SEM (*n* = 5 in each group). ^##^*p* < 0.01 vs. the untreated group or HJ901 group; *p < 0.05 and **p < 0.01 vs. the IMQ or CpG group.

In the SEAP assay, imiquimod (IMQ) and CpG 685 enhanced SEAP activity in HEK-Blue-hTLR7 and HEK-Blue-hTLR9 cells, whereas their activity was effectively reduced by HJ901 post-treatment in a dose-dependent manner (Figures 1A,B). The inhibitory effect of HJ901 on SEAP activity was reduced with increasing doses of IMQ or CpG 685 (Figures 1C,D). Unexpectedly, different effects were observed with HJ901 pre-treatment or post-treatment at different time points. As shown in Figures 1E–H, pre-treatment with HJ901 significantly inhibited SEAP activity in HEK-Blue-hTLR7 or HEK-Blue-hTLR9 cells at several different time points (0, 2, 4, 6, and 12 h) after exposure to IMQ or CpG 685. In contrast, when cells were pretreated with IMQ or CpG 685 over 2 h, the inhibition effect of HJ901 on the activity decreased. Moreover, we examined the cytotoxicity of HJ901 to HEK-Blue Null1, HEK-Blue hTLR 7, and HEK-Blue hTLR9 cells and observed no cytotoxicity toward these cells (Supplementary Figure S1).

### HJ901 Specifically Suppressed TLR7- and TLR9- Mediated Cell Proliferation and Reduced Cytokine Production in PBMCs

**FIGURE 2:**
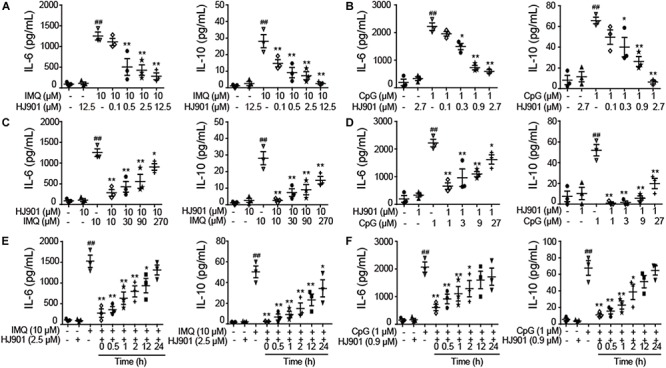
Suppressive effect of HJ901 on TLR7/9-mediated cytokine production in human PBMCs. **(A,B)** Human PBMCs were cultured with 10 μM IMQ or 1 μM CpG in the presence or absence of different concentrations of HJ901 (0.1, 0.5, 2.5, or 12.5 μM or 0.1, 0.3, 0.9, or 2.7 μM) for 36 h. **(C,D)** Human PBMCs were cultured with HJ901 in the presence or absence of different concentrations of IMQ (10, 30, 90, or 270 μM) or CpG (1, 3, 9, or 27 μM) for 36 h. **(E,F)** Human PBMCs were incubated with 10 μM IMQ or 1 μM CpG for 0, 0.5, 1, 2, 12, or 24 h and then treated with or without HJ901 for 36 h. Supernatants were analyzed for IL-6 and IL-10 levels with a cytometric bead array (CBA). Similar results were obtained from three independent experiments. All data are presented as the means ± SEM (*n* = 5 in each group). ^##^*p* < 0.01 vs. the untreated group or HJ901 group; *p < 0.05 and **p < 0.01 vs. the IMQ or CpG group.

To investigate whether HJ901 is a specific antagonist of TLR7/9, Poly (I: C) (a ligand of TLR3), lipopolysaccharide (LPS, a ligand of TLR4), imiquimod (IMQ, an agonist of TLR7), or CpG 685 (CpG, an agonist of TLR9) was used to induce increased cell proliferation in human PBMCs. However, IMQ and CpG 685 only promoted CD19^+^ B cell proliferation (Figure 1I). In contrast, HJ901 treatment effectively reduced CD19^+^ B cell proliferation stimulated by IMQ and CpG 685. However, the inhibitory effect of HJ901 was not exhibited in Poly (I: C)- and LPS-induced CD4^+^ T or CD8^+^ T cell proliferation analysis (Supplementary Figure S2), indicating that HJ901 specifically suppressed TLR7- and TLR9-mediated cell proliferation in human PBMCs. IMQ and CpG 685 induced cell proliferation in human PBMCs; 10 μM IMQ and 1 μM CpG 685 increased cell proliferation up to 41.6% and 82.8%, respectively (Supplementary Figure S3). However, this phenomenon was effectively blocked by HJ901 treatment in a dose-dependent manner (Figure 1J). Furthermore, we examined the effect of IMQ or CpG on TLR7/9-mediated cell proliferation inhibited by HJ901 in human PBMCs. The results showed that the ability of HJ901 to inhibit cell proliferation was decreased with increasing IMQ or CpG concentrations (Supplementary Figure S4). Additionally, HJ901 treatment remarkably reduced IL-6 and IL-10 generation caused by IMQ or CpG 685 in a dose- and time-dependent manner (Figures 2A–F).

### The Inhibitory Effect of HJ901 on Cell Proliferation and Cytokine Secretion in DLBCL Cell Lines

**FIGURE 3:**
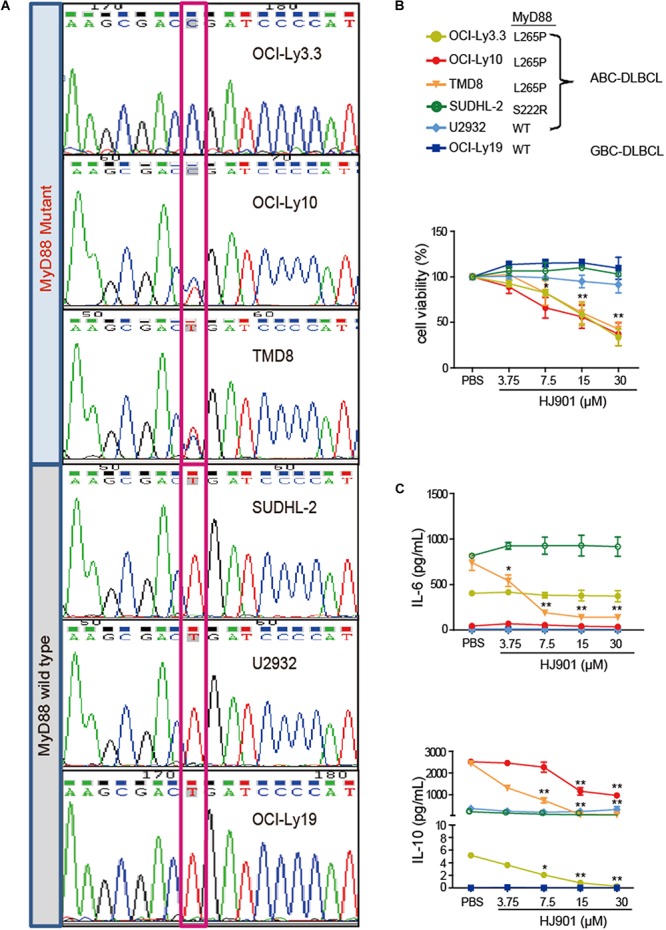
Inhibitory effect of HJ901 on cell proliferation and cytokine secretion *in vitro*. **(A)** Confirmation of MyD88 mutation by Sanger sequencing in cell lines. The position of the MYD88 L265P locus is indicated by black frame. **(B)** Cells were plated in 96-well plates and treated with or without HJ901 for 72 h. The cell proliferation percentage was determined by the WST-1 assay. **(C)** Cells were incubated in the presence or absence of HJ901 concentrations for 36 h. Cell supernatants were assessed for IL-6 and IL-10 levels by CBA. All data are presented as the means ± SEM (*n* = 5 in each group). *p < 0.05 and **p < 0.01 vs. PBS group.

The MyD88 gene in the OCI-Ly3.3, OCI-Ly10, TMD8, SUDHL-2, U2932, and OCI-Ly19 cell lines was sequenced by Sanger sequencing. Our results showed that serial dilution of DNA isolated from the OCI-Ly3.3 DLBCL cell line was homozygous for MyD88 L265P (T→C); OCI-Ly10 and TMD8 were heterozygous for MyD88 L265P, and the SUDHL-2, OCI-Ly19 and U2932 cells expressed the wild-type MyD88 gene (Figure 3A). Moreover, to confirm whether the TLR7 and TLR9 proteins were expressed in these cells, their gene and protein expression levels were evaluated by quantitative real-time PCR (qRT-PCR) and flow cytometry. We found that TLR7 and TLR9 transcripts and proteins were present in these cell lines (Supplementary Figure S5). WST-1 assays showed that HJ901 treatment effectively decreased cell viability in the ABC-DLBCL cell lines OCI-Ly3.3, OCI-Ly10, and TMD8 in a dose-dependent manner but did not significantly affect the viability of SUDHL-2, U2932, and OCI-Ly19 cells (Figure 3B). Interestingly, HJ901 treatment reduced the secretion of IL-10 in the ABC-DLBCL cell lines OCI-Ly3.3, OCI-Ly10, and TMD8, but IL-6 was only inhibited in TMD8 cells (Figure 3C).

### HJ901 Treatment Decreased Tumor Growth in a DLBCL Xenograft Mouse Model

**FIGURE 4:**
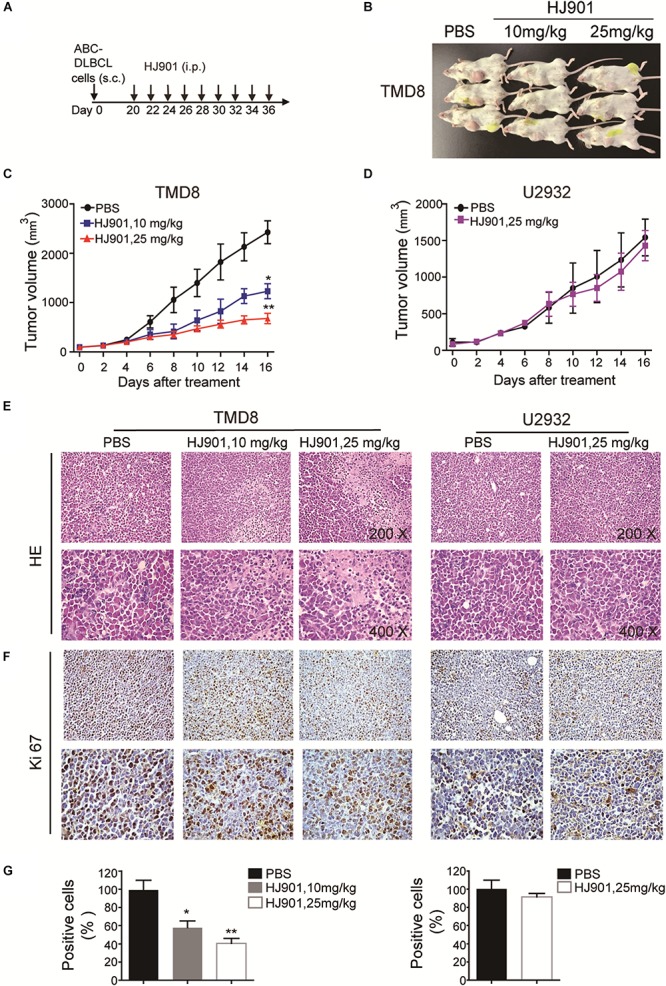
Clampdown effect of HJ901 on cell survival and cytokine secretion *in vivo*. **(A)** Mice with TMD8 or U2932 xenografts were treated with or without HJ901 (10 or 25 mg/kg). **(B)** The tumor size of mice injected with TMD8 was observed at day 36. **(C,D)** Tumor volumes were measured every other day for 16 days. **(E)** Representative histological sections of the tumors were stained with hematoxylin and eosin (H&E) (magnification × 200 and 400). **(F,G)** Immunohistochemistry was used to detect the expression of Ki67 in tumor specimens. Similar results were obtained from three independent experiments. All data are presented as the means ± SEM (*n* = 5 in each group). *p < 0.05 and **p < 0.01 vs. PBS group.

We also observed that HJ901 (10 or 25 mg/kg) treatment suppressed lymphoma growth in a mouse DLBCL xenogeneic tumor model with TMD8 cells harboring the MyD88 L265P mutation, rather than U2932 cells with wild-type MyD88 (Figures 4B–D). Histopathological and immune-histochemical analysis revealed that HJ901 treatment increased apoptotic and necrotic regions in the tumor tissues of mice with TMD8 cells compared to those in the control group. However, HJ901 treatment had no effect on tumor tissue in mice injected with U2932 cells (Figures 4E–G). These findings indicate that HJ901 treatment mainly inhibited tumor growth induced by cell lines expressing the MyD88 L265P mutation.

### HJ901 Treatment Arrested the Cell-Cycle in G1 Phase in Cell Lines Expressing the MyD88 L265P Mutation

**FIGURE 5:**
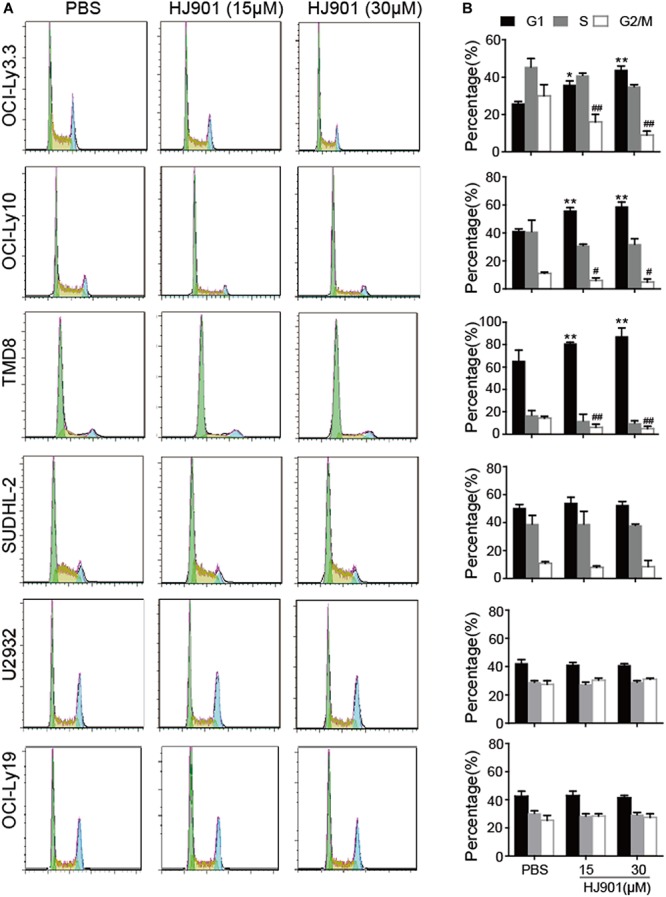
Effect of HJ901 on cell-cycle in DLBCL cell lines. All cells were incubated with or without HJ901 (15 or 30 μM) for 72 h. **(A,B)** The cell cycle was analyzed by propidium iodide staining and flow cytometry. Similar results were obtained from three independent experiments. All data are presented as the means ± SEM (*n* = 5 in each group). *p < 0.05 and **p < 0.01 vs. PBS group (G0/G1 phase); ^#^*p* < 0.05 and ^##^*p* < 0.01 vs. PBS group (S phase).

As illustrated in Figure 5, HJ901 treatment significantly increased the number of cells in G1 and reduced those in G2/M phase in OCI-Ly3.3, OCI-Ly10, and TMD8 cells but not in SUDHL-2, U2932, and OCI-Ly19 cells compared to the control treatment groups. We observed no change in S phase in any of the cell lines tested. Flow cytometry analysis of the different cell lines showed that apoptosis was not decreased by HJ901 treatment (Supplementary Figure S6). These results suggest that HJ901 treatment inhibited tumor growth by inhibiting cell proliferation rather than by promoting cell apoptosis.

### HJ901 Treatment Inhibited NF-κB and JAK2-STAT3 Pathway Activation *in vitro* and *in vivo*

**FIGURE 6:**
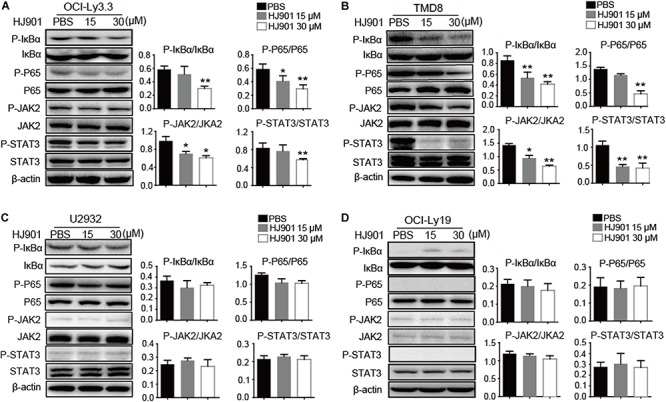
Inhibitory effect of HJ901 on NF-κB and JAK2-STAT3 pathways *in vitro*. **(A–D)** All cells lines were treated with or without HJ901 (15 or 30 μM) for 24 h and cell lysates were prepared for analysis of P-NF-κB/NF-κB, P-IκBα/IκBα, P-JAK2/JAK2, and P-STAT3/STAT3 by western blotting. The effect of HJ901 on the expression of P-NF-κB/NF-κB, P-IκBα/IκBα, P-JAK2/JAK2, and P-STAT3/STAT3 proteins are shown for these cell lines. Quantification of relative protein expression was performed by densitometric analysis using β-actin as an internal control. Similar results were obtained from three independent experiments. All data are presented as the means ± SEM (*n* = 5 in each group). *p < 0.05 and **p < 0.01 vs. PBS group.

**FIGURE 7:**
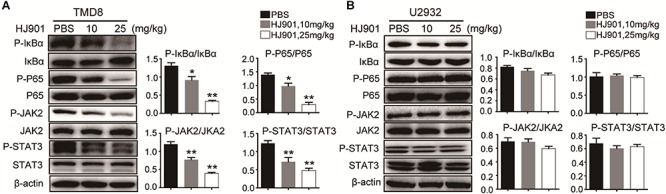
Suppressive effect of HJ901 on NF-κB and JAK2-STAT3 pathways *in vivo*. Mice with TMD8 or U2932 xenografts were treated with or without HJ901 (10 or 25 mg/kg). Tumor tissues were collected and analyzed by western blotting. Tumor tissues from TMD8 **(A)** and U2932 **(B)** xenografts mice with or without HJ901 treatment were collected and analyzed for the expression of P-NF-κB/NF-κB, P-IκBα/IκBα, and P-JAK2/JAK2, P-STAT3/STAT3 by western blotting.

We further evaluated the ability of HJ901 to inhibit NF-κB and JAK2-STAT3 activation *in vitro* and *in vivo* system. Our results revealed induction of NF-κB (P65), IκBα, JAK2, and STAT3 phosphorylation in OCI-Ly3.3 and TMD8 cells expressing the MyD88 L265P mutation. This induction was inhibited by HJ901 treatment. In contrast, these signaling pathways were not activated in OCI-Ly19 and U2932 cells expressing wild-type MyD88 (Figures 6A–D). Moreover, the expression of BTK and p38 phosphorylation did not change in any of the cell lines after HJ901 treatment (Supplementary Figure S7). Parallel studies were performed in a mouse xenograft model with TMD8 or U2932 ABC-DLBCL cell lines. As shown in Figures 7A,B, western blot analysis revealed that HJ901 treatment inhibited the induction of NF-κB (P65), IκBα, JAK2, and STAT3 phosphorylation in the mouse xenograft model when TMD8 cells with the MyD88 L265P mutation were used. However, these effects of HJ901 treatments were not observed in mice injected with U2932 cells expressing wild-type MyD88.

## Discussion

Uncontrolled activation of TLRs, including TLR7/9, results from somatic acquisition of gain-of-function mutations in MyD88, which affects many hematological malignancies (Ngo et al., 2011). ABC-DLBCL is characterized by frequent accumulation of MyD88 mutations and constitutively activated NF-κB and JAK2-STAT3 signaling pathways (Davis et al., 2001; Ding et al., 2008; Lam et al., 2008b). Consequently, targeting proteins associated with these pathways is a promising treatment for ABC-DLBCL. ODN possess a multitude of biological activities (Zhu et al., 2002; Shirota et al., 2004; Dong et al., 2005). In this study, HJ901, a synthetic ODN containing a sequence with CCT repeats, was found to exhibit a specific inhibitory role in TLR7/9 activation-induced innate immune responses and in reducing cell proliferation and tumor growth in ABC-DLBCL cell lines expressing the MyD88 L265P mutation.

Increasing evidence has indicated that DLBCL is an aggressive disease accompanied by a high proliferation rate (Blonska et al., 2015). In this study, HJ901 treatment significantly reduced cell proliferation not only in human PBMCs treated with IMQ or CpG 685 but also in ABC-DLBCL cells OCI-Ly3.3, OCI-Ly10, and TMD8 with the MyD88 L265P oncogenic mutation confirmed by Sanger sequencing. In addition, in a xenograft mouse model of DLBCL induced by TMD8 cells with the MyD88 L265P mutation, we found that HJ901 treatment effectively inhibited tumor growth by inducing cell apoptosis and inhibiting Ki67 expression in tumor tissues, which is the hallmark of growth suppression measured by immune histochemical analysis (Jin et al., 2016). In addition, flow cytometry analysis of apoptosis and cell-cycle detection showed that HJ901 reduced cell proliferation by arresting cells in G1 and G2/M phases rather than causing apoptosis in OCI-Ly3.3, OCI-Ly10, and TMD8 cells, indicating that its anti-tumor mechanism in intact animals is more complex than *in vitro*. These observations suggest that HJ901 efficiently inhibited cell proliferation and tumor growth *in vitro* and *in vivo*.

Moreover, several reports have also revealed that cytokines affect tumor cells in many hematologic malignancies (Weidle et al., 2010; Grivennikov and Karin, 2011). Indeed, IL-10 and/or IL-6 signaling in ABC-DLBCL tumors regulate the malignant cell and tumor microenvironment (Wang et al., 2004; Lam et al., 2008b; Wang et al., 2014), suggesting that suppression of IL-10 and IL-6 secretion is beneficial for treating ABC-DLBCL. Our results indicate that HJ901 treatment not only effectively decreased the production of IL-10 and IL-6 in dose- and time-dependent manners, but also suppressed IL-6 and IL-10 generation in cells treated with increasing doses of IMQ or CpG 685, indicating that HJ901 competitively combines with TLR7 and TLR9 receptors.

Multiple signal pathways are involved in the pathogenesis of ABC-DLBCL. Constitutive NF-κB activity plays a significant role in promoting cell proliferation and survival in ABC-DLBCL (Davis et al., 2001). Furthermore, NF-κB has an important effect on ABC-DLBCL biology in that it induces cytokine IL-6 and IL-10 production (Ding et al., 2008; Lam et al., 2008b). Our western blot analysis revealed that HJ901 treatment can inhibit NF-κB (P65) and IκBα phosphorylation in OCI-Ly3.3 and TMD8 cells with the MyD88 L265P mutation but not in wild-type MyD88-expressing OCI-Ly19 and U2932 cells. In the mouse xenograft model with TMD8 or U2932 ABC-DLBCL tumor induction, HJ901 treatment also blocked NF-κB signaling activation with TMD8. Previous reports showed that TLR-mediated NF-κB activation could be evaluated by detecting SEAP activity (Zhang et al., 2018). In this study, we found that HJ901 effectively reduced SEAP activity in HEK-Blue-hTLR7 or HEK-Blue-hTLR9 cells treated with IMQ or CpG685, further indicating that HJ901 effectively inhibits TLR7/TLR9 and suppresses TLR-mediated NF-κB activation.

However, a recent study reported a high level of STAT3 expression and activation in ABC-DLBCL cell lines (Ding et al., 2008). Additionally, there is constitutively activated JAK-STAT3 signaling and generation of IL-6 and IL-10 in cells expressing the MyD88 L265P mutation (Ngo et al., 2011). In turn, secretion of these cytokines further results in JAK-STAT3 signaling activation as part of an autocrine loop that promotes the survival and proliferation of lymphoma cells (Lam et al., 2008b; Ngo et al., 2011). The current results suggest that phosphorylation of JAK2 and STAT3 was significantly down-regulated after HJ901 treatment in cell lines and mice harboring the MyD88 L265P mutation. Collectively, HJ901 treatment may have reduced IL-6 and IL-10 secretion in cells and suppressed cell proliferation and tumor growth in cells and mice through possible mechanisms that modulate the TLR7/9, NF-κB, and JAK2-STAT3 signaling pathways.

In summary, HJ901, which is an antagonist of TLR7 and TLR9, not only specifically and effectively inhibited TLR7- and TLR9-mediated cell proliferation, but also inhibited IL-6 and IL-10 secretion *in vitro*. Moreover, HJ901 treatment significantly reduced tumor growth in a DLBCL xenograft mouse model with the MyD88 L265P mutation. Our results further indicate that these mechanisms are involved in the down-regulation of NF-κB and JAK2-STAT3 signaling pathways *in vivo* and *in vitro*. Accordingly, the suppressive ODN HJ901 may target the TLR7/9-mediated signaling pathway and be useful as a novel strategy for treating patients with ABC-DLBCL expressing the MyD88 L265P mutation.

## Materials and Methods

### Oligonucleotides and Reagents

Nuclease-resistant phosphorothioate-modified ODNs were synthesized by Suzhou Ribo Life Science Co., Ltd. (Suzhou, China). The Poly (I: C) (a ligand of TLR3), lipopolysaccharide (LPS, *Escherichia coli* 055: B5, a ligand of TLR4), imiquimod (IMQ, a ligand of TLR7), and CpG 685 (CpG, a ligand of TLR9) were obtained from InvivoGen (San Diego, CA, United States). Fetal bovine serum (FBS) was purchased from Gibco (Grand Island, NY, United States). Penicillin and streptomycin, carboxyfluorescein succinimidyl amino ester (CFSE), and RPMI-1640 medium were purchased from Invitrogen (Carlsbad, CA, United States). APC-CD3, V450-CD4, Percpcy-5.5-CD8, APC-H7-CD19, anti-rabbit IgG, and appropriate isotype-controls, the Cytometric beads array kit (human IL-6/-10 Flex set), and Annexin V/PI apoptosis kit were obtained from BD Biosciences (Franklin Lakes, NJ, United States). The WST-1 Cell Proliferation Assay Kit was provided by the Beyotime Institute of Biotechnology (Jiangsu, China). Antibodies against P-P65 (serine 536)/P65, P-IκBα/IκBα, P-JAK2 (tyrosine 1008)/JAK2, P-STAT3 (tyrosine 705)/STAT3, and β-actin were provided by Cell Signaling Technology (Danvers, MA, United States) or Santa Cruz Biotechnology (Dallas TX, United States). The catalog numbers and dilution ratios of all antibodies are listed in Table 1.

**TABLE 1:** Catalog numbers for antibodies.

**Antibodies**	**Product**	**Source**	**Catalog No.**	**Dilution**
CD3	APC Mouse Anti-Human CD3	BD Pharmingen	555335	2 μL/10^6^ cells
CD4	V450 Mouse Anti-human CD4	BD Pharmingen	560345	2 μL/10^6^ cells
CD8	PerCP-Cy5.5 Mouse Anti-Human CD8	BD Pharmingen	565310	2 μL/10^6^ cells
CD19	APC-H7 Mouse Anti-Human CD19	BD Pharmingen	560727	3 μL/10^6^ cells
Carboxyfluoresceinsuccinimidyl amino ester (CFSE)	CellTrace CFSE Cell Proliferation Kit	Invitrogen	C34554	2 μL/10^6^ cells
Aqua	LIVE/DEAD Fixable Aqua Dead Cell Stain Kit	Invitrogen	L34597	1 μL/10^6^ cells
TLR7	Human TRL7 PE-conjugated antibody	R&D	IC5875P LOT:ABEV0312071	5 μL/10^6^ cells
TLR7 isotype control	PE anti-mouse IgG2a	Biolegend	407108 LOT:B182280	2 μL/10^6^ cells
TLR9	APC Rat anti-human CD289	BD Pharmingen	560428	3 μL/10^6^ cells
TLR9 isotype control	Rat IgG2a K Isotype	eBioscience	4299699	2 μL/10^6^ cells
P-IκBα	P-IκBα (Ser-32) Rabbit mAb	CST	2859	1:1000
T-IκBα	IκBα(C-21)	Santa Cruz	sc-371	1:500.
P-P65	P-NF-kB p65 (serine 536) Rabbit mAb	CST	3033	1:1000
T-P65	NF-kB p65 Rabbit mAb	CST	4764	1:2000
P-JAK2	P-Jak2 (tyrosine 1008) Rabbit mAb	CST	8082S	1:1000
JAK2	Jak2 XP Rabbit mAb	CST	3230S	1:1000
P-STAT3	P-Stat3(tyrosine 705) Mouse mAb	CST	9138	1:1000
STAT3	Stat3 Mouse mAb	CST	4904	1:2000
P-p38	Phospho-p38 MAPK (Thr180/Tyr182) Rabbit mAb	CST	4631S	1:1000
T-p38	p38 MAPK Antibody	CST	9212S	1:1000
P-Btk	Phospho-Btk (Tyr223) Antibody	CST	5082S	1:1000
T-Btk	Btk (C82B8) Rabbit mAb	CST	3533S	1:1000
β-actin	Mouse monoclonal	CST	2242334	1:1000
Ki-67 primary antibodies	Ki-67/MKI67 Antibody		Novusbio	1:100
IL-6	Hu IL-6 CBA Flex Set A7	BD Pharmingen	558276	1:250
IL-10	Hu IL-10 CBA Flex Set B7	BD Pharmingen	558274	1:250

### Cell Culture

Human peripheral blood mononuclear cells (hPBMCs) were isolated from buffy coats of healthy blood donors (The Blood Center of Jilin Province, Changchun, China) by Ficoll-Hypaque (GE HealthCare, Little Chalfont, United Kingdom) density gradient centrifugation. The hPBMCs were washed three times with RPMI-1640 medium. All participants were volunteers who provided informed consent for use of their data for research purposes. The protocol used was approved by the institutional ethics committee (approval No.: 2017-031). Viability of hPBMCs was determined by trypan blue exclusion to be 95–99%. These cells were cultured in RPMI 1640 medium supplemented with 10% (v/v) heat-inactivated FBS and antibiotics (100 IU penicillin/mL and 100 IU streptomycin/mL). As shown in Table 2, the human DLBCL-derived cell lines OCI-Ly3.3, TMD8, OCI-Ly10, SU-DHL2, U2932, and OCI-Ly19 were obtained from different laboratories. All cell lines were cultured in Iscove’s modified Dulbecco’s medium (IMDM, Hyclone, Logan, UT, United States) with 20% (v/v) heat-inactivated FBS, β-mercaptoethanol (55 μM, Invitrogen) and antibiotics at 37°C in a 5% CO_2_ humidified incubator. All cell lines were confirmed to have the MYD88 L265P mutation by Sanger DNA sequencing.

**TABLE 2:** Characteristics of cells used in the study.

**Subtype**	**Cell line**	**MyD88 status**	**Source**
ABC	OCI-Ly 3.3	L265P Homozygous	Provided by Dr. Marc Minden*
	OCI-Ly 10	L265P Heterozygous	Provided by Dr. Marc Minden*
	TMD8	L265P Heterozygous	Provided by Dr. Liguang Lou**
	SUDHL-2	WT (S222R mutation)	ATCC***
	U2932	WT	DSMZ****
GCB	OCI-Ly 19	WT	Provided by Dr. Marc Minden*

*Dr. Marc Minden: University Health Network, Toronto, Canada. **Dr. Liguang Luo: Shanghai Institute of Materia Medica, Chinese Academy of Sciences, Shanghai 201203, China ***ATCC: American Type Culture Collection ****DSMZ: The Deutsche Sammlung von Mikroorganismen und Zellkulturen (Leibniz-Institut DSMZ, Braunschweig, Germany) *****OCI-Ly3.3, OCI-Ly10, OCI-Ly19 and TMD8 were identified by short tandem repeat.

### Cytokine Assays

Human PBMCs (5 × 10^5^ cells/well) from healthy volunteer blood were isolated and cultured with 10 μM IMQ or 1 μM CpG 685 in the presence or absence of different concentrations of HJ901 for 36 h. Next, the cell supernatants were analyzed for cytokine IL-6 and IL-10 with a cytometric beads assay kit (CBA; BD Biosciences).

### Cell Proliferation Assay

Cell viability was determined by a WST-1 assay following the manufacturer’s instructions. The cells were seeded into 96-well plates for 12 h and cultured with different doses of HJ901 (0, 3.75, 7.5, 15, and 30 μM) for 72 h. Subsequently, the cells were used in the WST-1 assay for 2 h to evaluate cell proliferation. The proliferation of human PBMCs was monitored by CFSE dilution assay via flow cytometry. The PBMCs (5 × 10^5^ cells/well) labeled with CFSE were plated into 96-well U-bottomed plates and cultured with 1 μg/mL Poly (I: C), 5 μg/mL LPS, 10 μM IMQ, or 1 μM CpG 685 in the presence or absence of HJ901 for 5 days. The cells were harvested and stained with Aqua (Invitrogen), APC-CD3, APC-H7-CD19 anti-rabbit IgG, and appropriate isotype controls. Proliferation of PBMCs was monitored with a CFSE dilution assay using a Fortessa flow cytometer (BD Biosciences) and Cell Quest^TM^ software. Data were analyzed using FlowJo V10 analysis software (BD Biosciences).

### Secreted Embryonic Alkaline Phosphatase (SEAP) Assay

Human embryonic kidney (HEK) 293 cells stably expressing human TLR7 or TLR9 cell lines, HEK-Blue TLR7, HEK-Blue TLR9, and its parental cell line HEK-blue Null1 cells were obtained from Invivogen (San Diego, CA, United States) and used to access TLR-mediated NF-κB activation by measuring the SEAP activity (Zhang et al., 2018). Briefly, 3 × 10^5^ cells/mL HEK-Blue TLR7, HEK-Blue TLR9, or HEK-blue Null1 cells were seeded into 96-well plates for 48 h and then treated with 10 μM IMQ or 1 μM CpG685 in the presence or absence of different concentrations of HJ901. Additionally, the cells were treated with HJ901 in the presence or absence of various concentrations of IMQ or CpG685. These cells were also incubated with 10 μM IMQ or 1 μM CpG685 for 0, 2, 4, 6, and 12 h, and subsequently treated with or without HJ901. At the end of treatment, 20 μL of the culture supernatant was removed from each treatment and tested for SEAP activity using 180 μL of Quanti-Blue substrate following the manufacturer’s protocol (Invivogen). TLR7 or TLR9-mediated NF-κB activation can be assessed by measuring SEAP activity.

### DNA Sequencing of MyD88 Variants

Genomic DNA was extracted from 3 × 10^6^ OCI-Ly3.3, OCI-Ly10, TMD8, SUDHL-2, and OCI-Ly19 cells using a genomic DNA kit (Trans Gen Biotech Co., Beijing, China) and the product of the MyD88 gene sequence was amplified using Tks Gflex DNA Polymerase (Takara Biotechnology, Shiga, Japan) and PCR. The following forward and reverse primers were used for MYD88: 5′-GTTGAAGACTGGGCTTGTCC-3′ and 5′-AGGAGGCAGGGCAGAAGTA-3′ (Poulain et al., 2013). Amplified PCR products were extracted using a gel extraction kit (Kangwei Biotechnology, Beijing, China). We transformed this product using the pEasy-Blunt cloning kit (Trans Gen Biotech Co.) and the purified PCR products were directly sequenced for MyD88 by the Genwiz Co. (Beijing, China). The following thermal cycler parameters were used: 95°C for 30 min, 30 cycles of 95°C for 10 s, 60°C for 10 s, and 68°C for 30 s, and followed by 68°C for 5 min. Data were analyzed with Seq Scape software version 2.5 (Applied Biosystems, Foster City, CA, United States).

### Mouse and Animal Protocols

Six to eight-week-old female NOD-SCID mice weighing 18–22 g were raised by the Vital River Laboratory Animal Technology Co., Ltd., (Beijing, China) and were maintained in a pathogen-free animal facility at the Institute of Translational Medicine, The First Hospital, Jilin University. All studies were performed in accordance with the institutional guidelines and the protocols were approved by the ethics committee of the First Hospital of Jilin University, Changchun, China (approval No.: 2018-041). To induce tumor formation, mice were subcutaneously injected in the right flank with TMD8 or U2932 (1 × 10^7^) cells. Briefly, as shown in Figure 4A, the tumor volumes were measured every other day with Vernier calipers for 16 days and were calculated as *V* = (a × b^2^)/2, where “a” is the length of tumor and “b” is the width of tumor. When the tumor volume reached 80–120 mm^3^, the mice were intraperitoneally injected with 10 or 25 mg/kg HJ901 three times/week or with PBS, which served as a control.

### Cell Cycle Analysis

Various cells were plated into 6-well plates and incubated with or without 15 or 30 μM HJ901. After 72 h, the cells were harvested, washed twice with PBS, and then subjected to cell cycle analysis following our previously published protocol (Niu et al., 2018).

### Western Blotting

Protein lysates were prepared from U2932, TMD8, OCI-Ly19, and OCI-Ly3.3 cells treated with 0, 15, and 30 μM of HJ901 for 24 h. Western blot analysis was performed based on our previously published methods (Sundaramoorthy et al., 2013). Specific primary antibodies, including P-P65/P65, P-IκBα/IκBα, P-JAK2/JAK2, P-STAT3/STAT3, and β-actin, were detected by western blotting. The experiments were repeated three times for each experimental condition.

### Histopathological and Immunohistochemistry Analysis

Fresh tumor tissues were collected, immediately fixed, and embedded in paraffin. Next, the tumor tissues were cut into 5-μm-thick sections and stained with hematoxylin-eosin (H&E) staining to assess pathological changes in the tumors by light microscopy. Additionally, fixed slides were incubated in 3% H_2_O_2_ for 10 min to suppress endogenous peroxidase activity and then washed in PBS. Next, the slides were blocked with 1.5% serum in PBS at room temperature for 30 min followed by 5% BSA for 20 min. Slides were incubated with Ki-67 primary antibodies (Novus Biologicals, Littleton, Co., United States) overnight at 4°C. After overnight incubation, the slides were washed with PBST for 15 min and secondary antibodies tagged with biotin were added for 90 min and incubated with Strept Avidin-Biotin Complex (SABC) (Boster, Wuhan, China). After incubating the slides with a solution of 3,3′-diaminobenzidine tetrahydrochloride (Boster), the tissue was checked under a microscope to confirm appropriate staining. The slides were then counterstained, dehydrated, cleared, and mounted. Positively stained cells were counted using Motic Images Advanced 3.2 (Media Cybernetics, Rockville, MD, United States).

### Statistical Analysis

All data referenced above were presented as the means ± SEM and were analyzed using SPSS19.0 (SPSS, Inc., Chicago, IL, United States). Comparisons between experimental groups were conducted using one-way analysis of variance, whereas multiple comparisons were made using the least significant difference method. The comparative CT method was applied in the qRT-PCR assay according to the delta-delta CT method. Statistical significance was defined as *p* < 0.05 or *p* < 0.01.

### Ethics Approval and Consent to Participate

Human and Animal experiments were approved by the ethics committee of the First Hospital of Jilin University, Changchun, China (approval No.: 2017-031 and 2018-041, respectively).

## Data Availability Statement

The raw data analyzed during the current study are available from the corresponding author upon reasonable request.

## Ethics Statement

The studies involving human participants were reviewed and approved by the ethics committee of the First Hospital of Jilin University. The patients/participants provided their written informed consent to participate in this study. The animal study was reviewed and approved by the ethics committee of the First Hospital of Jilin University.

## Author Contributions

BA and JtC initiated the study. BA, SZ, TL, JW, GZ, XL, and YQ performed the experiments and analyzed the data. TL and XL contributed to animal studies. JH, YS, and JwC contributed to the discussion. Y-JL and JtC conceived and designed the experiments and supervised the work. All authors reviewed and edited the manuscript.

## Conflict of Interest

YS was employed by the Changchun Huapu Biotechnology Company.

The remaining authors declare that the research was conducted in the absence of any commercial or financial relationships that could be construed as a potential conflict of interest.
