# Supplementary material for: A Dual TLR7/TLR9 Inhibitor HJ901 Inhibits ABC-DLBCL Expressing the MyD88 L265P Mutation
Source: Front Cell Dev Biol. 2020 Apr 22;8:262. doi: 10.3389/fcell.2020.00262 (PMC7188833; doi:10.3389/fcell.2020.00262)

**SUPPLEMENTAL DATA**

**Materials and methods**

**Oligonucleotides and reagents**

Nuclease-resistant phosphorothioate-modified ODNs and Con ODN (5′-tggccaagcttgggccccttgcaagggcc-3′) were synthesized by the Huapu Co. (Shanghai, China). The imiquimod (TLR7 L) and CpG 685 (TLR9 L) were purchased from Invivogen (San Diego, CA, USA). Fetal bovine serum (FBS) was purchased from Gibco (Grand Island, NY, USA). Penicillin and streptomycin, carboxyfluorescein succinimidyl amino ester (CFSE), and RPMI-1640 medium were purchased from Invitrogen (Carlsbad, CA, USA). APC-CD3, V450-CD4, Percpcy-5.5-CD8, APC-H7-CD19, anti-rabbit IgG, and appropriate isotype-controls were obtained from BD Biosciences (Franklin Lakes, NJ, USA). Antibodies against P-BTK (tyrosine 223)/BTK, P-P38 (threonine180/tyrosine182)/P38, and β-actin were obtained from Cell Signaling Technology (Danvers, MA, USA) or Santa Cruz Biotechnology (Dallas, TX, USA).

**Cell culture**

Human peripheral blood mononuclear cells (hPBMCs) were isolated from buffy coats of healthy blood donors (The Blood Center of Jilin Province, Changchun, China) by Ficoll-Hypaque (GE Healthcare, Little Chalfont, UK) density gradient centrifugation. PBMCs were washed three times with RPMI-1640 medium. The viability of the PBMCs was determined to be 95–99% by trypan blue exclusion. The isolated cells were cultured in RPMI-1640 medium supplemented with 10% (v/v) heat-inactivated FBS and antibiotics (100 IU/mL penicillin and 100 IU/mL streptomycin). As shown in **Table 2**, the human DLBCL-derived cell lines OCI-Ly3.3, TMD8, OCI-Ly10, SUDHL-2, U2932, and OCI-Ly19 were obtained from different laboratories. All cell lines were cultured in Iscove's modified Dulbecco's medium (IMDM, Hyclone, Logan, UT, USA) with 20% (v/v) heat-inactivated FBS, β-mercaptoethanol (55 μM, Invitrogen) and antibiotics (100 IU/ml penicillin and 100 IU/ml streptomycin) at 37°C in a 5% CO_2_ humidified incubator.

**Cell proliferation assay**

The proliferation of human PBMCs was determined by CFSE labeling with flow cytometry. The PBMCs (5 × 10^5^ cells/well) were labeled with CFSE and plated into 96-well U-bottomed plates and cultured with different IMQ (0.1, 0.5, 1, 5, or 10 μM) or CpG685 (0.1, 0.5, 1, 5, or 10 μM) concentrations for 5 days. The cells were harvested and stained with Aqua, APC-CD3, V450-CD4, Percep-cy5.5-CD8, anti-rabbit IgG, and appropriate isotype-controls, respectively. The proliferation of PBMCs was determined using the mean fluorescent intensity of CFSE-labeled cells by BD FACS (LSRII, BD Biosciences), and the data were analyzed using FlowJo software.

**Measurement of TLR7 and TLR9 in tumor cell lines**

For quantitative real-time PCR (qRT-PCR), total RNA was prepared from all cells using Trizol reagent and a Prime-Script RT-PCR kit (Takara, Shiga, Japan). The following PCR primer sequences (forward and reverse) were used to amplify TLR9: 5′-TGAAGACTTCAGGCCCAACTG-3′ and 5′-TGCACGGTCACCAGGTTGT-3′; TLR7: 5′-TTACCTGGATGGAAACCAGCTACT-3′ and 5′-TCAAGGCTGAGAAGCTGTAAGCTA-3′; and GAPDH: 5′- GAAGGTGAAGGTCGGAGTC-3′; and 5′-GAAGATGGTGATGGGATTTC-3′. Data analysis was performed using the delta CT method. For flow cytometry analysis, cells were stained with anti-TLR7-PE and anti-TLR9-APC antibodies.

**Apoptosis assay**

OCI-Ly3.3, OCI-Ly10, TMD8, SUDHL-2, U2932, and OCI-Ly19 cells were cultured in 12-well plates (5 × 10^5^ cells/well) for 24 h and treated with 15 or 30 μM HJ901 for an additional 72 h. The cells were harvested and centrifuged at 600×*g* for 5 min at 4°C. Next, the cells were stained with FITC-labeled Annexin V and propidium iodide. Cell numbers were analyzed using FlowJo software (LSRII Flow Cytometery; BD Biosciences).

**Western blotting**

Protein lysates were prepared from OCI-Ly3.3, TMD8, U2932, and OCI-Ly19 cells treated with different doses of HJ901 (0, 15 and 30 μM) for 24 h. Western blot analysis was performed based on our previously published methods ([Sundaramoorthy et al., 2013](#_ENREF_30)). Then, specific primary antibody, including P-BTK/BTK, P-P38/P38, and β-actin were detected by western blotting. The experiments were repeated three times under each experimental condition.

**Statistical analysis**

All data are presented as the means ± SEM and analyzed using SPSS19.0 (SPSS, Inc., Chicago, IL, USA). Comparisons between experimental groups were conducted using one-way analysis of variance, whereas multiple comparisons were made using the least significant difference method. The comparative CT method was applied in the qRT-PCR assay according to the delta-delta CT method. Statistical significance was defined as p < 0.05 or p < 0.01.

**Supplemental Figure legends**

**FIGURE S1**. **Effect of HJ901 on HEK-Blue Null1, HEK-Blue hTLR7, and HEK-Blue hTLR9 cells.** (A) HEK-Blue-Null1 cells were cultured with PBS, Con ODN, or 1 μM CpG685 in the presence or absence of different concentrations of HJ901 (0.001, 0.002, 0.5, 2.5, or 12.5 μM) for 24 h and the secreted embryonic alkaline phosphatase (SEAP) activity was determined. (B and C) The cytotoxicity of H901 to HEK-Blue-hTLR7 or HEK-Blue-hTLR9 cells. The cells were cultured with PBS, Con ODN, 10 μM IMQ, 1 μM CpG685 or in the different concentrations of HJ901 (0.001, 0.02, 0.5, 2.5, or 12.5 μM) for 24 h and SEAP activity was determined.


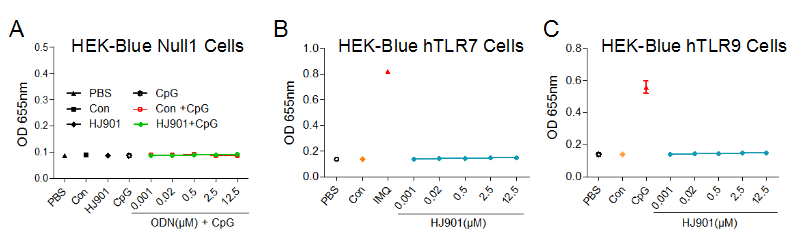


**FIGURE S2. Effect of HJ901 on TLR7/9-mediated cell proliferation in human PBMCs.**(**A–B**) Human PBMCs incorporated CFSE cultured with Poly (I: C), LPS, IMQ, or CpG685 (CpG) in control ODN, and HJ901 cells for 5 days of treatment. Proliferation of CD4^+^ and CD8^+^ cells was determined by CFSE dilution assessed by flow cytometry. Similar results were obtained from three independent experiments. All data are presented as the means ± SEM (n = 5 in each group).


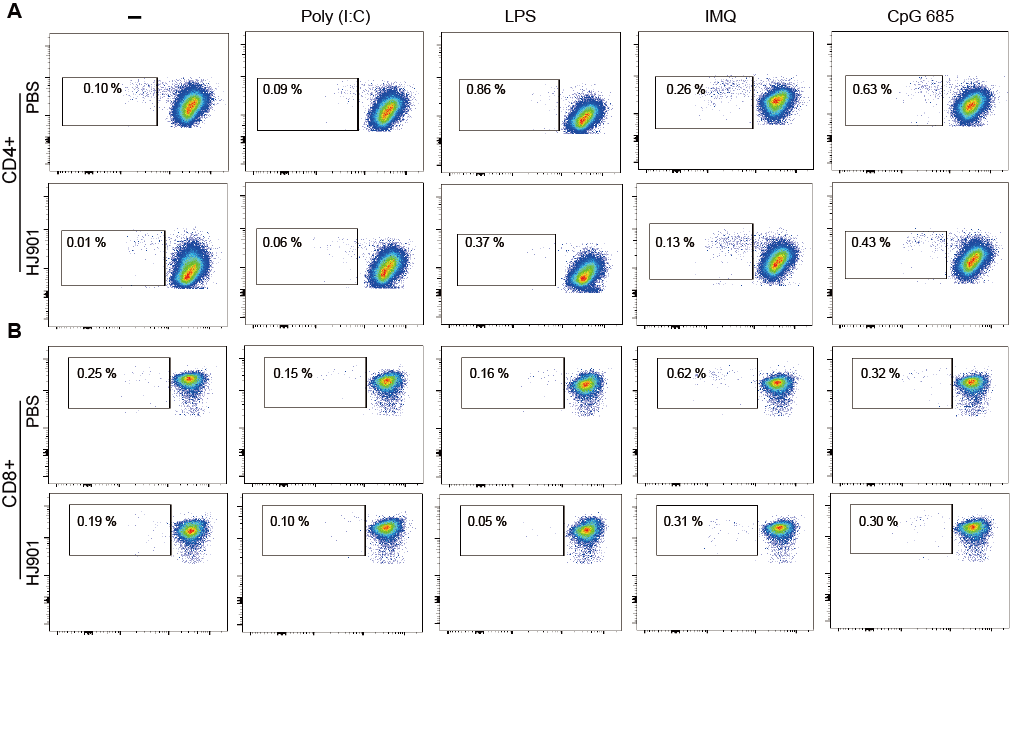


**FIGURE S3. Effect of IMQ and CpG685 on proliferation of human PBMCs.** Human PBMCs with incorporated CFSE were cultured with different concentrations of IMQ (0.1, 0.5, 1, 5, or 10 μM) or CpG685 (0.1, 0.5, 1, 5or 10 μM) for 5 days. The CD19^+^ cell proliferation was determined by CFSE dilution, which was assessed by flow cytometry. Similar results were obtained from three independent experiments. All data are presented as the means ± SEM (n = 5 in each group).


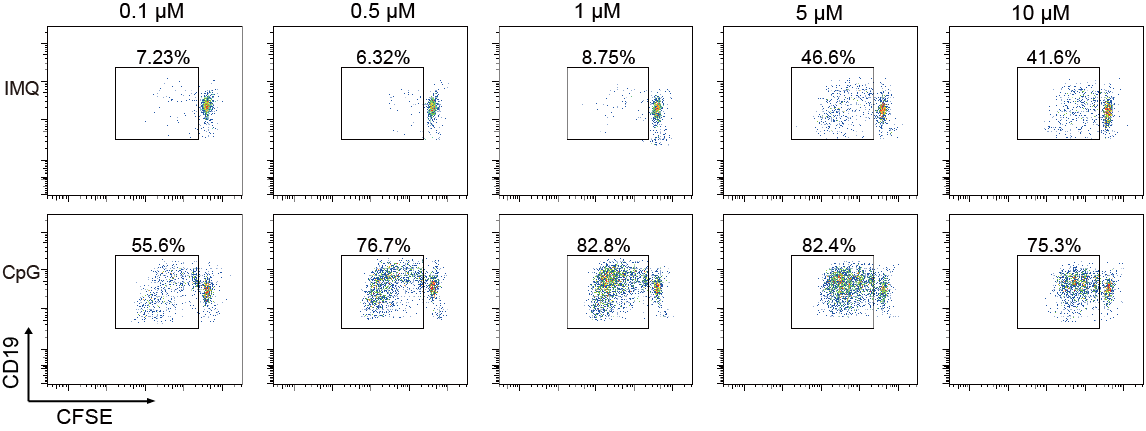


**FIGURE S4. Effect of IMQ or CpG685 on TLR7/9-mediated cell proliferation inhibited by HJ901 in human PBMCs.** Human PBMCs were cultured with HJ901 in the presence or absence of different concentrations of IMQ (10, 30, 90 or 270 μM) or CpG685 (1, 3, 9 or 27 μM) for 5 days. Proliferation of CD19^+^ B cell were determined by CFSE dilution, which was assessed by flow cytometry. Quantification of three experiments is shown in the right panel. Similar results were obtained from three independent experiments. All data are presented as the means ± SEM (n = 5 in each group). ^##^*p* < 0.01 vs. untreated group or HJ901 group; ^*^*p* < 0.05 and ^**^*p* < 0.01 vs. IMQ or CpG group.


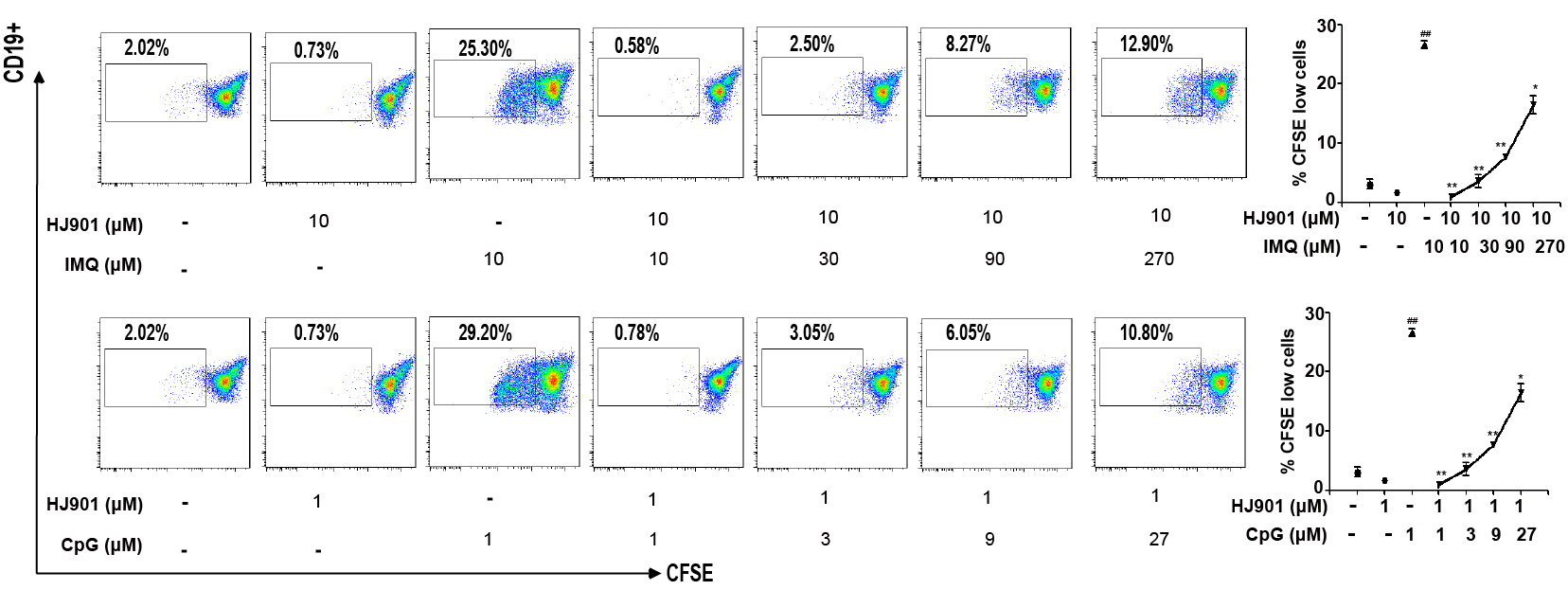


**FIGURE S5. Expression of TLR7 and TLR9 in DLBCL cell lines.** (**A–B**) Expression of intracellular TLR7 and TLR9 in cell lines by qRT-PCR analysis. (**C–D**) Cells were stained with anti-TLR7-PE, anti-TLR9-APC antibodies (white histograms), and isotype controls (gray histograms) were determined by flow cytometry.


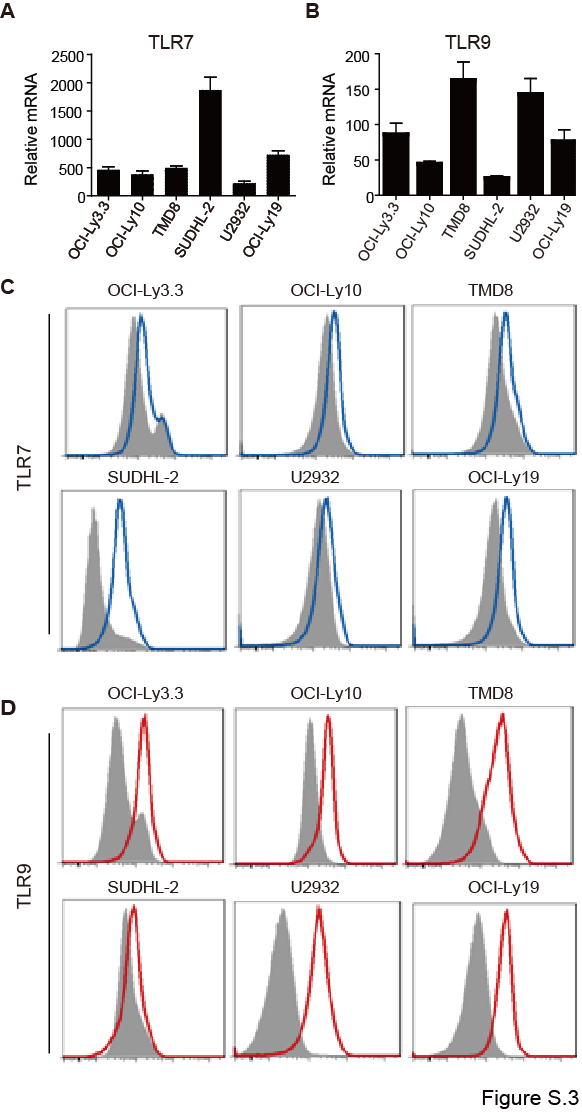


**FIGURE S6. Effect of HJ901 on cell apoptosis in DLBCL cell lines.** Cells were plated in 12-well plates and treated with or without HJ901 (15 or 30 μM) for 72h and stained with AnnexinV and propidiumiodide to evaluate apoptosis by flow cytometry. Similar results were obtained from three independent experiments. All data are presented as the means ± SEM (n = 5 in each group).


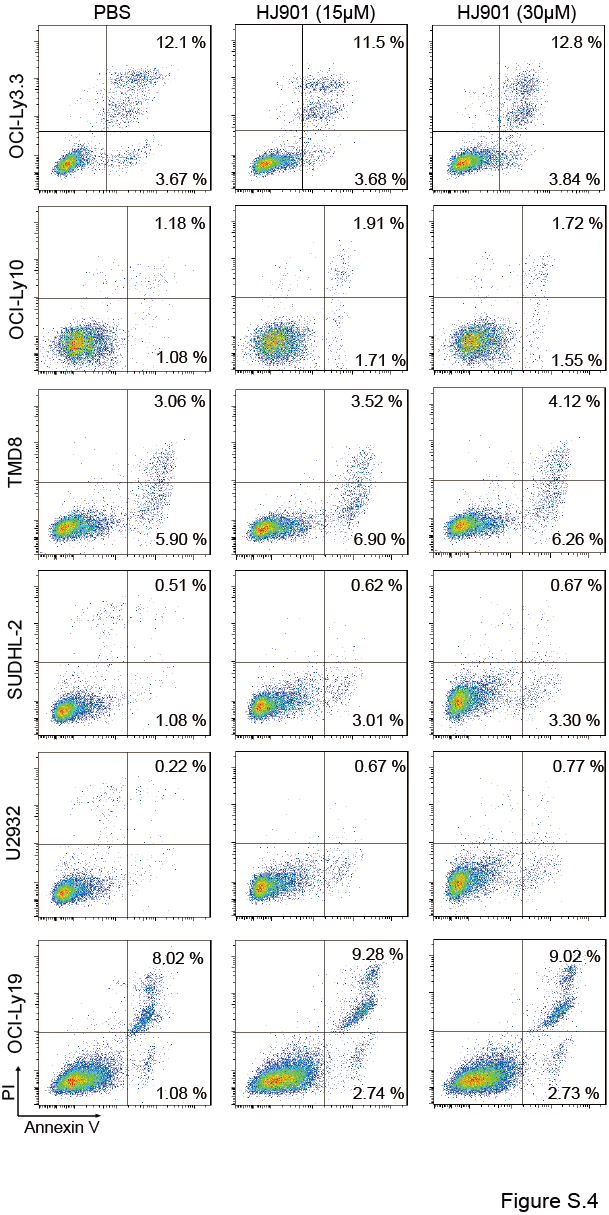


**FIGURE S7. Effect of HJ901 on BTK and P38 signaling in DLBCL cell lines.** All cell lines were treated with or without HJ901 (15 or 30 μM) for 24 h. **(A–D)** Cell lysates were prepared for analysis with P-BTK/BTK, and P-P38/P38 by western blotting. Relative protein expression was quantified by densitometric analysis. Similar results were obtained from three independent experiments. All data are presented as the means ± SEM (n = 5 in each group).


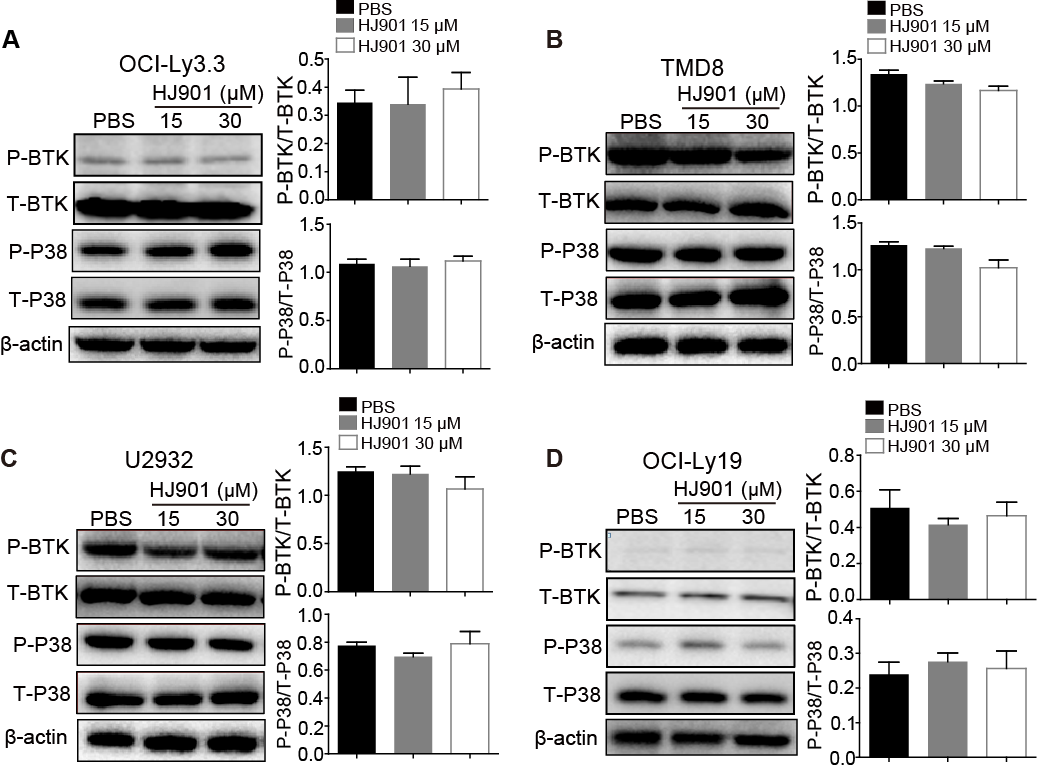

Supplement: Supplementary file 1 [file Data_Sheet_1.docx]
